# Retraction: The impact of COVID-19 and muscle fatigue on cardiorespiratory fitness and running kinetics in female recreational runners

**DOI:** 10.3389/fphys.2026.1793994

**Published:** 2026-02-06

**Authors:** Frontiers Editorial Office

Following publication, concerns were raised regarding the ethical approval procedure, recruitment, and methodology of the study that failed to adequately ensure the safety of previously hospitalised COVID-19 patients. An investigation was conducted in accordance with Frontiers’ policies.

The authors failed to provide a satisfactory explanation, and, as a result, the conclusions of the article have been deemed unreliable, the study has been found not to meet appropriate ethical standards, and the article has been retracted.

This retraction was approved by the Chief Editors of Frontiers in Physiology and the Chief Executive Editor of Frontiers. The authors disagree to this retraction.

